# The effects of feeding benzoic acid and/or active dry yeast (*Saccharomyces cerevisiae*) on fatty acid composition, sensory attributes, and retail shelf-life of beef *longissimus thoracis*

**DOI:** 10.1093/tas/txac161

**Published:** 2022-12-07

**Authors:** Melissa S Williams, Ira B Mandell, Katharine M Wood, Benjamin M Bohrer

**Affiliations:** Department of Animal Biosciences, University of Guelph, Guelph, Ontario, N1G 2W1, Canada; Department of Animal Biosciences, University of Guelph, Guelph, Ontario, N1G 2W1, Canada; Department of Animal Biosciences, University of Guelph, Guelph, Ontario, N1G 2W1, Canada; Department of Animal Sciences, The Ohio State University, Columbus, Ohio 43210, USA

**Keywords:** benzoic acid, fatty acid composition, feed additives, meat quality, *Saccharomyces cerevisiae*, shelf-life

## Abstract

Fifty-nine Angus-cross steers (492 ± SD 36 kg) were arranged in a randomized complete block design and assigned to the following dietary treatments for the final 106 days of the finishing phase: no supplementation (**CON**), 0.5% benzoic acid (**ACD**), 3 g/steer/d active dry *Saccharomyces cerevisiae* (**YST**), or both [0.5% benzoic acid and 3 g/steer/d *S. cerevisiae* (**AY**)]. Steers were slaughtered at a commercial facility where *longissimus thoracis* (IMPS #107 Beef Rib) samples were retrieved and evaluated for fatty acid composition, sensory attributes, and shelf-life during a simulated retail display period. Data (*N* = 57) were analyzed using dietary treatment as a fixed effect, blocking weight at the beginning of the study as a random effect, and steer as the experimental unit. Muscle pH and proximate composition (moisture and intramuscular lipid) for *longissimus* samples were not different (*P* ≥ 0.39) among dietary treatments. Most fatty acid profile values and calculations were not different among dietary treatments (*P* ≥ 0.10); however, the *n*-6:*n*-3 ratio differed (*P* = 0.01), with ACD samples having lower *n*-6:*n*-3 compared with CON and YST samples while AY samples were intermediate and not different from other dietary treatments. The trained sensory panel did not detect differences among dietary treatments (*P* ≥ 0.23) for juiciness, beef flavor intensity, or off-flavor intensity; however, they did score AY samples as chewier than ACD samples with CON and YST samples intermediate and not different from other dietary treatments. Yet, tenderness was not different when scored by trained panelists (*P* = 0.10) or measured instrumentally (*P* = 0.21). Total color change tended to differ (*P* = 0.09) during the 12-d simulated retail display period with AY samples experiencing less color change compared with YST samples, while CON and ACD samples were intermediate and not different from other dietary treatments. Lipid oxidation (as measured with TBARS) tended to differ (*P* = 0.08) following the 12-d simulated retail display period with ACD and AY samples experiencing lower levels of oxidation compared with CON, while YST samples were intermediate and not different from other dietary treatments. Overall, these results suggest there were no negative impacts on meat quality when finishing steers were supplemented with either benzoic acid or *S. cerevisiae*, and there may even be advantages for fatty acid composition and oxidative stability when steers were supplemented with benzoic acid.

## INTRODUCTION

Due to evolving concerns for antibiotic-resistance and consumer demands, livestock producers are often asked to raise animals without the use of antibiotics. In response to these interests, natural feed additive products with antimicrobial effects have been marketed as alternatives to the antibiotics traditionally found in livestock rations and are gaining attention among animal science researchers. In addition to understanding how novel feed additives improve animal performance, health, and carcass traits, it is also essential to expand this research with focus on how these additives may impact meat quality, meat merchandising ability, and consumer eating experience.

Over the past several decades, organic acids have been used in a variety of livestock feeding programs to serve as preservatives and acidifiers (Martin, 1998; Papatsiros et al., 2012; Pearlin et al., 2020). Organic acids, such as formic acid, lactic acid, acetic acid, sorbic acid, propionic acid, and benzoic acid, exert antimicrobial effects by suppressing fungal activity and maintaining a homeostatic rumen environment, and in some cases this has led to measurable beneficial effects on growth performance and health status (Freitag, 2007; Tugnoli et al., 2020). Benzoic acid is a weak organic acid (pKa = 4.19) with an aromatic ring structure that has shown promising effects as a feed ingredient for beef cattle (Wang et al., 2020a, 2020b) as well as other meat-producing livestock species such as swine (Chen et al., 2017; Diao et al., 2016; Zhai et al., 2017) and poultry (Józefiak et al., 2007, 2010; Weber et al., 2012). When compared with other organic acids which have an aliphatic ring structure and primarily represent a source of energy for cells, benzoic acid has different metabolic and absorption characteristics (Chipley, 2020; Mao et al., 2019). Benzoic acid has been shown to exhibit unique effects on intracellular membrane activity in the presence of fungal microorganisms (Arroyo-López et al., 2008; Hazan et al., 2004; Warth, 1988). However, it should be noted that studies evaluating the interactive effects of benzoic acid and fungal microorganisms have been primarily limited to the food and beverage industry and very little research has been conducted on livestock feeds and feeding to this point. Direct-fed microbials, such as active yeast products, are used to increase the proportion of beneficial microbes in an animal’s gastrointestinal system which helps mitigate pathogens and enhance digestive efficiency (Krehbiel et al., 2003; Seo et al., 2010; McAllister et al., 2011). Specifically in beef cattle, supplementation with active yeast products has the potential to improve feedlot performance, as well as reduce the shedding of harmful microbials in feces (Brashears et al., 2003; Krehbiel et al., 2003; Stephens et al., 2010).

To-date, there has been limited research conducted on meat quality, meat merchandising ability, and consumer eating experience for beef products from cattle supplemented with benzoic acid, active dry yeast, or their combination. Therefore, the objectives of this study were to examine the effects of feeding cattle a high-grain finishing diet supplemented with benzoic acid, active dry yeast (*Saccharomyces cerevisiae*), or a combination of benzoic acid and active dry yeast on the fatty acid composition, sensory attributes, and shelf-life of *longissimus thoracis* samples.

## MATERIALS AND METHODS

All animal procedures in this study were approved by the University of Guelph Animal Care Committee (Animal Utilization Protocol #3706). Animals were received and managed in accordance with the Animal Utilization Protocol, which was approved based on guidelines and principles of the Canadian Council on Animal Care (1993).

All procedures in this study that involved human participants (sensory testing) were approved by the University of Guelph Human Ethics Committee REB Project #17-12-017. Written, informed consent was obtained from each participant before the start of screening and training for the sensory evaluations.

### Live Animal Procedures

Live animal procedures were fully described by Williams et al. (2021). To summarize, 59 Angus-cross steers were used in the feeding trial associated with this study. Steers were housed inside a covered finishing barn in six 7.16 × 14.07 m pens for the duration of the study. Each pen was equipped with four Insentec feeding stations (Insentec B.V., Marknesse, the Netherlands) that enabled the measurement of individual steer feed intakes and multiple treatment diets to be provided to each pen; therefore, individual steer was considered as the experimental unit for analyses. Steers were blocked by starting body weight (BW) into three groups (light: 370–416 kg, intermediate: 421–443 kg, and heavy: 445–527 kg), which determined the pen assignments. Within each block (two pens per block), steers were equally and randomly assigned to one of four dietary treatment groups, with each pen containing two dietary treatments assigned to four or five steers in that pen (total of nine or ten steers per pen).

Steers were fed high-grain diets consisting of 79.3–79.8% high moisture corn 10% alfalfa haylage, 10.2–10.7% soybean meal, dietary treatment ingredients (benzoic acid and/or active dry yeast) and a premix containing 33 mg/kg monensin, vitamins, and minerals. The dietary treatment ingredients replaced a portion of the high-moisture corn in the diet and were added with the soybean meal and premix. There were four dietary treatments in this study for the final 106 days of finishing: 1) no benzoic acid or active dry yeast added to the finishing diet (**CON**, *n* = 15 steers), 2) supplementation with benzoic acid (Vevovitall, DSM Nutritional Products, Parsippany, New Jersey, USA) at 0.5% on a DM basis (**ACD**, *n* = 14 steers), 3) supplementation with active dry yeast (*S. cerevisiae*, Vistacell; AB Vista, Marlborough, UK) at 3 g per steer per day resulting in 60 billion colony forming units (CFU) per steer per day (**YST**, *n* = 15 steers), and 4) supplementation with both benzoic acid at 0.5% and active dry yeast (*S. cerevisiae*) at 3 g per steer per day (**AY**, *n* = 15 steers).

### Sample Collection

On the morning of day 107 of the study (after 106 days on the dietary treatment diets), steers were transported a short distance (approximately 30 km) to a commercial meat processing facility. Steers were humanely handled and slaughtered (*via* captive bolt stunning, followed by exsanguination) using commercial industry standards under the inspection of the Canadian Food Inspection Agency (CFIA). Individual animal ID was maintained throughout the slaughter process. Carcass sides were ribbed by plant personnel, and *longissimus thoracis* rib portions anterior to the 12th and 13th rib interface (IMPS #107 Beef Rib) were collected and delivered to the University of Guelph Meat Laboratory. Fifty-seven of the 59 samples were delivered to the University of Guelph Meat Laboratory and used for this study (CON, *n* = 15 samples; ACD, *n* = 14 samples; YST, *n* = 15 samples; AY, *n* = 13 samples).

At 6 days post-mortem, beef ribs were boned, trimmed of excess fat, and the ribeye cap (*spinalis dorsi*) was removed. The remaining portions (*i.e.*, *longissimus thoracis* muscle samples) were cut into 2.5-cm thick steaks beginning at the posterior end (*i.e.*, 12th and 13th rib interface) and assigned the following analyses: steak #1—pH and proximate composition; steak #2 and steak #3—instrumental tenderness; steak #4 and steak #5—shelf-life during a simulated retail display period; steak #6—fatty acid analysis; and steak #7–trained sensory analysis.

### pH and Proximate Composition

Ultimate pH (6 days post-mortem) for *longissimus thoracis* samples (*i.e.*, steak #1) was measured in triplicate using a calibrated spear-tipped pH meter (Hanna HI98163; Hanna Instruments, Mississauga, Ontario, Canada). Following the pH measurement, samples were vacuum packaged and stored at ≤ −22 °C until moisture and lipid composition of *longissimus thoracis* muscle was determined. Samples were first thawed in a refrigerator overnight (15–20 h), trimmed of external subcutaneous fat, cubed, and homogenized using a counter-top food processor (KitchenAid model KHB23511CU; St. Joseph, Missouri, USA). Duplicate 5 g samples of the homogenate were weighed onto an aluminum weighing dish and covered with two #1 Whatman Qualitative filter papers (42 mm; GE Healthcare Life Sciences; Chicago, Illinois, USA). Next, the samples were dried in a Fisherbrand Isotemp drying oven (Thermo Fisher Scientific; Ottawa, Ontario, Canada) at 100 °C for at least 24 h and then weighed again to determine moisture by subtraction (method 950.46; AOAC, 2007). The dried samples were then placed in the Soxhlet extraction apparatus and washed multiple times over a 5-h time period using approximately 200 mL of warm petroleum ether. Washed samples were placed into the 100 °C drying oven for a minimum of 24 h to evaporate the petroleum ether, and then weighed for calculation of lipid by subtraction (method 991.36; AOAC, 2007).

### Fatty Acid Analysis

*Longissimus thoracis* samples assigned for fatty acid analysis (*i.e.*, steak #6) were vacuum packaged at 6 days post-mortem and stored at ≤ −22 °C until analysis took place. When analysis was set to take place, samples were thawed in a refrigerator overnight (15–20 h), trimmed of external subcutaneous fat, cubed, and homogenized using a food processor (KitchenAid model KHB23511CU; St. Joseph, Missouri, USA). Fatty acid composition was determined *via* lipid extraction based on the method of Bligh and Dwyer (1959) in the presence of known amounts of internal standard for total lipid/fatty acid analyses. Internal standards were obtained from NuChek Prep (Elysian, Minnesota, USA). An aliquot of the total lipid extract was collected to quantify the fatty acids following transmethylation (Morrison and Smith, 1964). The fatty acid methyl esters were prepared using boron trichloride in methanol and by heating the methylation tubes to 95 °C in a water bath. The fatty acid methyl esters were analyzed using an Agilent 7890B gas-liquid chromatograph (Santa Clara, California, USA) with a 60-meter DB-23 capillary column (0.32 mm internal diameter) and using standard mixtures (qualitative and quantitative) with the known fatty acid components for verification obtained from American Oil Chemists Society (AOCS, Champaign, Illinois, USA) and Sigma–Aldrich (St. Louis, Missouri, USA).

### Sensory Testing

*Longissimus thoracis* samples assigned for sensory testing (*i.e.*, steak #7) were vacuum packaged at 6 days post-mortem, wet-aged until 14 days post-mortem, and then stored at ≤ −22 °C until analysis took place. Sensory panels for this study were held in conjunction with those in the Dorleku et al. (2021) study, so methodology used in this study mirror those described in that study. In summary, screening and training of panelists were conducted in accordance with standards provided by the American Meat Science Association (AMSA, 2016). Panelists were introduced and rigorously trained on each of the sensory parameters that were evaluated and then screened based on their abilities immediately prior to the beginning of sensory testing. Screened and selected panelists consisted of 14 individuals between the ages of 18 and 30. These panelists were trained over 2 weeks (10 training sessions) for the evaluation of tenderness, chewiness, juiciness, beef-flavor intensity, and off-flavor using a 15 cm line scale with anchors at 0, 7.5, and 15 cm.

A total of seven sensory evaluation sessions were held over seven consecutive weekdays. Frozen vacuum-packaged steaks were thawed for 24 h at approximately 4 °C prior to being cooked to an overall internal temperature of 72 °C using an indoor grill (model No. 25360, Hamilton Beach; Markham, Ontario, Canada). Eight cooked steaks (two steaks from each of the four dietary treatments) were trimmed of their outside edges, cut into 1 cm cubes, and then served to panelists in a randomized order. Each panelist was served two cubes from each steak at ambient temperature and humidity in a 29.5 mL capped plastic cup. Panelists were seated in individual booths under overhead red lighting to prevent visual bias. Each panelist was served the samples in random order along with bottled water and unsalted crackers, which served as palate cleansers. Panelists were instructed to cleanse their palates between samples. Panelists scored samples on a 15 cm line scale for tenderness (where 0 indicated extremely tough and 15 indicated extremely tender), chewiness (where 0 indicated not chewy and 15 indicated extremely chewy), juiciness (where 0 indicated not juicy and 15 indicated very juicy), beef flavor intensity (where 0 indicated very weak beef flavor and 15 indicated very intense beef flavor), and off-flavor intensity (where 0 indicated no off-flavor detected and 15 indicated very intense off-flavor). All responses were collected and recorded using Compusense version 5.8 software (Guelph, Ontario, Canada).

### Instrumental Tenderness

*Longissimus thoracis* samples assigned for instrumental tenderness testing (*i.e.*, steak #2 and steak #3) were vacuum packaged at 6 days post-mortem, wet-aged until either 7 days post-mortem (steak #2) or 14 days post-mortem (steak #3), and then stored at ≤ −22 °C until analysis took place. Warner–Bratzler shear force (WBSF) was determined using methodology previously described by Streiter et al. (2012). Briefly, steaks were thawed and weighed before being cooked to an internal temperature of 72 °C on a clamshell Garland Grill (Ed-30B: Garland Commercial Ranges LTD, Mississauga, Ontario, Canada) set to a surface temperature of 105 °C. Following cooking, samples were cooled in a refrigerator to an internal temperature of approximately 4 °C. Six to eight 1.25-cm diameter cores running parallel to the muscle fibers were removed from each steak. Each core was sheared perpendicularly to the muscle fibers with a Warner–Bratzler blade using a TA-XT Plus Texture Analyzer (Texture Technologies Corp., Scarsdale, New York, USA) at a crosshead speed of 3.3 mm/s. The average peak force was recorded for each *longissimus thoracis* sample.

### Simulated Retail Display

*Longissimus thoracis* samples assigned for simulated retail display testing (*i.e.*, steak #4 and steak #5) were collected at 6 days post-mortem. One sample (*i.e.*, Steak #4) was evaluated using the thiobarbituric acid reactive substances (TBARS) assay at the beginning of the simulated retail display period. One sample (*i.e.*, Steak #5) was used to evaluate color stability during the simulated retail display period with TBARS determined at the end of the simulated retail display period.

### Assessment of Color Stability

The sample used to evaluate color stability (and TBARS at the end of the simulated retail display period) was placed directly on top of a meat soaker pad (Tite-Dri Industries, Boynton Beach, Florida, USA) on a styrofoam tray and then overwrapped with 60-gauge meat wrapping film (Western Plastics, Calhoun Georgia, USA) using an Avantco WM-18 single roll film wrapping machine (Avantco Equipment, USA). The overwrapped trays were laid out onto two multi-level meat display cases. Each tier of the display case was separated at an equal distance, and each level was illuminated with two 1.22 m long LED lights (52 W, 1850 lumens, 1612.5–2152 lux). Trays were shuffled once every 24 h such that an even amount of illuminance was applied to all samples over the display period. Instrumental color and surface discoloration (% metmyoglobin formation) were evaluated daily until the study population reached an average surface discoloration of 60%, which coincided with day 12 of the display period. Surface discoloration (%) was evaluated by two trained panelists on each day of the shelf-life study using Meat Color Measurement Guidelines outlined by AMSA (2016) and a visual discoloration scoring standard previously outlined by Wang et al. (2020b). Instrumental color was evaluated using a calibrated, handheld Minolta CR-400 Chroma meter (Konica Minolta Sensing Americas, Inc, Ramsey, New Jersey, USA) with illuminant D_65_ and 0° viewing angle settings. As per the Commission International de l’Eclairage (CIE, 1976), each measurement using the Chroma meter was reported using the L*, a*, and b* color space. Two measurements per sample were collected and then averaged to determine instrumental color values for each sample. Chroma, a measure of color intensity, was calculated using the equation:$\sqrt{{\left(a*\right)}^{2}+\;{\left(b*\right)}^{2}}$. Hue angle, a measure of distance in degrees from the true red axis of the CIE color space, was calculated using the formula: tan^−1^$\left(\frac{b*}{a*}\right)$. Delta *E* 76 (ΔE*_ab_), a measure of total color difference, was calculated for the difference between instrumental color on the first and last day of the display period using the equation:


$$\begin{array}{l} \sqrt{{\left(Time\;\;2\;\;L*\;-\;\;Time\;\;1\;\;L*\right)}^{2}}\\ +\;{\left(Time\;\;2\;\;a*\;\;-\;\;Time\;\;1\;\;a*\right)}^{2}\\ +\;{\left(Time\;\;2\;\;b*\;-\;Time\;\;1\;\;b*\right)}^{2} \end{array}$$

At the end of the study, the samples were vacuum packaged and stored ≤ −22 °C until further analysis (*i.e.*, the TBARS assay using the methodology outlined below) was performed.

### Assessment of Lipid Oxidation

The TBARS assays were performed using a slightly modified version of the method described by Leick et al. (2010) and the same methodology previously described by Wang et al. (2020b) and Dorleku et al. (2021). Samples were trimmed of external subcutaneous fat, and ground in a food processor to obtain a homogenous sample. The ground meat samples were blended with 1 mL of butylated hydroxytoluene (BHT) and 45.5 mL of 10% trichloroacetic acid in 0.2 M phosphoric acid (TCA/H_3_PO_4_) using a Waring industrial blender (Conair Corporation, Stamford, Connecticut, USA). The blended sample was then filtered using filter paper (No. 1 Whatman; GE Healthcare Life Sciences, Kent, UK) into two 5 mL duplicates. Five milliliters of thiobarbituric acid (TBA) were then added to one of the two duplicates creating a test sample and a blank sample. The samples were then incubated for 16 h in the dark at room temperature. Samples were then assessed for malondialdehyde (MDA) content using a 96-well plate in a plate reader (Synergy HT, BioTek Instruments, Inc, Winooski, Vermont, USA) at 530 nm wavelength. A standard concentration curve was plotted with 1,1,3,3-tetraethoxypropane (TEP) to determine MDA concentration. Samples were corrected using recovery rate percentages captured using spiked samples which consisted of 1 mL of 0.2 mg/mL of BHT, 12 mL of TEP, and 32 mL of TCA/H_3_PO_4_. Spiked samples were prepared in duplicate at the same time as test samples. Both spiked samples and test samples were tested in duplicate, and results were expressed as mg MDA/kg of meat.

### Statistical Analysis

Data (*N* = 57) were analyzed as a randomized complete block design with dietary treatment as a fixed effect, blocking weight at the beginning of the finishing period as a random effect, and steer as the experimental unit. An additional random effect of day and panelist was included for the sensory data and an additional random effect of the day was included for the color evaluation. All data were analyzed using the GLIMMIX procedure in SAS version 9.4 (SAS Institute Inc. Cary, North Carolina, USA). Least squares means were separated using the PDIFF option in SAS and a Tukey–Kramer adjustment was utilized to protect against committing a Type-I statistical error. Results were considered significant at *P* ≤ 0.05 and tendencies were considered at 0.05 < *P* < 0.10.

## RESULTS AND DISCUSSION

### pH and Proximate Composition

pH and proximate composition (moisture and intramuscular lipid) were unaffected (*P* ≥ 0.39) by dietary treatment (Table 1). The chemical assays used in this study confirmed the results for marbling of these cattle reported by Williams et al. (2021) which were not different among treatments and were as follows: CON = Small^75^; ACD = Small^75^; YST = Small^24^; AY = Small^36^. Wang et al. (2020a) previously reported no differences in muscle pH for steers supplemented with benzoic acid when compared to beef from steers-fed control diets; however, Wang et al. (2020a) did report greater levels of intramuscular lipid in *longissimus thoracis* samples from steers supplemented with benzoic acid compared with *longissimus thoracis* samples from steers fed control diets. Geng et al. (2016a) reported no differences in muscle pH for steers supplemented with active dry *S. cerevisiae* when compared with beef from steers fed control diets. Several studies (Geng et al., 2016a; Ovinge et al., 2018; Ran et al., 2018) have reported no differences in marbling in the *longissimus thoracis* for cattle supplemented with active dry *S. cerevisiae* when compared with beef from cattle-fed control diets. The lack of response for intramuscular lipid in the present study is not surprising as metabolic precursors for marbling, such as circulating content of glucose and non-esterified fatty acids, were unaffected by the treatment diets fed in this population of steers (Williams et al., 2021). However, previous studies have suggested that feeding live yeast has changed the proportions of ruminal volatile fatty acids in beef cattle (Armato et al., 2016; Cagle et al., 2020), which could alter the rate and quantity of gluconeogenesis occurring from a metabolic standpoint and therefore improve lipogenesis in intramuscular fat depots (Smith and Crouse, 1984; Schoonmaker et al., 2004).

**Table 1.** Proximate composition and fatty acid profile for beef *longissimus thoracis* from steers fed a high-grain finishing diet with no supplementation, benzoic acid, active dry yeast, or a combination of benzoic acid and active dry yeast

	Treatment^1^					
Item	CON	ACD	YST	AY	SEM	*P*-value
pH	5.45	5.44	5.45	5.42	0.02	0.39
Proximate composition						
Moisture, %	71.72	71.50	71.55	71.77	0.56	0.98
Intramuscular lipid, %	5.44	5.59	5.36	5.21	0.71	0.98
Fatty acid composition, mg/100 g sample						
Total SFA^2^	7,285	8,085	7,395	7,113	571	0.64
Total MUFA^3^	10,145	10,945	10,685	9,712	711	0.60
Total PUFA^4^	977	868	951	915	51	0.41
*n*-6 PUFA^5^	818	700	798	749	46	0.24
*n*-3 PUFA^6^	159	168	153	165	8	0.50
Fatty acid composition, % of total fatty acids						
Total SFA^2^	33.88	34.71	33.57	34.53	0.66	0.54
Total MUFA^3^	47.45	47.71	48.35	47.49	0.61	0.66
Total PUFA^4^	4.75	3.93	4.53	4.57	0.31	0.24
*n*-6 PUFA^5^	3.98	3.17	3.81	3.74	0.28	0.17
*n*-3 PUFA^6^	0.76	0.76	0.72	0.83	0.05	0.25
Fatty acid ratio						
MUFA:SFA^7^	1.41	1.38	1.45	1.38	0.04	0.62
PUFA:SFA^8^	0.14	0.11	0.14	0.13	0.01	0.23
PUFA:MUFA^9^	0.10	0.08	0.09	0.10	0.01	0.26
*n*-6:*n*-3 ratio^10^	5.27^a^	4.14^b^	5.28^a^	4.53^ab^	0.28	0.01

^a-b^ Least square means within a row with different superscripts differ (*P* < 0.05).

^1^ Treatments: CON (*n* = 15): control (not supplemented); ACD (*n* = 14): 0.5% of benzoic acid dietary inclusion on a DM basis (DSM Nutritional Products); YST (*n* = 15): 3g/hd/d of *Saccharomyces cerevisiae* (Vistacell, AB Vista, Marlborough, UK); AY (*n* = 13): 0.5% of benzoic acid dietary inclusion on a DM basis (DSM Nutritional Products) and 3 g/hd/d of *Saccharomyces cerevisiae* (Vistacell, AB Vista, Marlborough, UK).

^2^ Total saturated fatty acids (SFA) = C6:0 + C7:0 + C8:0 + C9:0 + C10:0 + C11:0 + C12:0 + C14:0 + C15:0 + C16:0 + C17:0 + C18:0 + C19:0 + C20:0 + C22:0 + C24:0.

^3^ Total monosaturated fatty acids (MUFA) = C12:1 + C14:1 + C15:1 + C16:1 + C17:1 + C18:1 + C19:1 + C20:1 + C22:1 + C24:1.

^4^ Total polyunsaturated fatty acids (PUFA) = C18:2 + C18:3 + C18:4 + C20:2 + C20:3 + C20:4 + C20:5 + C22:2 + C22:4 + C22:5 + C22:6.

^5^ Total *n*-6 polyunsaturated fatty acids.

^6^ Total *n*-3 polyunsaturated fatty acids.

^7^ MUFA:SFA = total monounsaturated fatty acids ÷ total saturated fatty acids.

^8^ PUFA:SFA = total polyunsaturated fatty acids ÷ total saturated fatty acids.

^9^ PUFA:MUFA = total polyunsaturated fatty acids ÷ total monounsaturated fatty acids.

^10^ n-6 fatty polyunsaturated fatty acids ÷ n-3 polyunsaturated fatty acids.

### Fatty Acid Analysis

The majority of the fatty acid composition values and calculations were not different (*P* ≥ 0.17) among the dietary treatments evaluated in this study (individual fatty acid values expressed in units of mg/100 g sample are presented in Supplementary Table S1 and individual fatty acid values expressed in units of percentage of total fatty acid are presented in Supplementary Table S2). However, the ratio of *n*-6:*n*-3 differed (*P* = 0.01) among dietary treatments. A lower ratio of *n*-6:*n*-3 is more favorable from a human nutrition perspective and ideally the ratio should be less than 4.0 (Scollan et al., 2005). In this study, the *n*-6:*n*-3 ratios for beef intramuscular fat were similar for steers supplemented with ACD (4.14) and AY (4.53). However, the *n*-6:*n*-3 ratios for beef intramuscular fat from steers fed CON (5.27) and steers supplemented with YST (5.28) were statistically greater (*P* < 0.05) than the *n*-6:*n*-3 ratio beef intramuscular fat from steers fed ACD (4.14). Changes in *n*-6:*n*-3 ratio of beef intramuscular fat have been shown to be influenced by several production factors including genetics, nutrition, and age of the animal; however, most researchers agree that the causal mechanism is variations in rumen biohydrogenation (Jenkins et al., 2008; Dewhurst and Moloney, 2013; Vahmani et al., 2015). Rumen biohydrogenation is the conversion of unsaturated fatty acids to saturated fatty acids that occurs in the rumen *via* microbial fermentation (Lourenço et al., 2010). To our knowledge, no previous research investigating benzoic acid on microbial fermentation or rumen biohydrogenation patterns has been initiated, thus this remains an area of research that should be highly prioritized in the future. The only other available study that has been completed to-date measuring fatty acid profile for beef from cattle supplemented with benzoic acid was Wang et al. (2020b), which reported no difference in *n*-6:*n*-3 of beef subcutaneous fat tissue from steers supplemented with benzoic acid when compared with control samples. Therefore, the results from this study should be evaluated in additional populations of cattle to increase the confidence of this meaningful finding.

It was surprising that greater differences in *n*-6:*n*-3 ratio of beef intramuscular fat were attributed to benzoic acid supplementation rather than yeast supplementation. A recent review conducted by Amin and Mao (2021) discussed yeast supplementation’s ability to influence biohydrogenation pathways in the rumen by providing a favorable environment or directly encouraging the growth of bacteria involved with the generation of conjugated linoleic acid and other biohydrogenation intermediates. However, the current study did not support these proposed effects as no differences in fatty acid composition values were detected between steers supplemented with YST when compared with steers fed the CON.

### Sensory Testing and Instrumental Tenderness

The trained sensory panel scored samples from steers supplemented with AY as chewier (*P* < 0.05) than samples from steers that were not supplemented (CON) or samples from steers supplemented with ACD, with samples from YST steers at an intermediate chewiness value that was not different from other dietary treatments (Table 2). Yet, tenderness was not different among dietary treatments when scored by the trained panelists (*P* = 0.10) or measured instrumentally (*P* = 0.21) at either 7 days of post-mortem aging or 14 days of post-mortem aging. In addition, the panelists did not detect differences (*P* ≥ 0.23) in juiciness, beef flavor intensity, or off-flavor intensity among the dietary treatments evaluated in this study. These findings conflict with previous findings published by Wang et al. (2020a), which reported that *longissimus thoracis* samples from steers supplemented with benzoic acid were more tender, less chewy, juicier, and more flavorful when compared with samples from steers fed control diets. One factor that should be considered from the Wang et al. (2020a) study was that samples in that study from steers supplemented with benzoic acid had greater intramuscular lipid content (6.41%) compared with samples from steers fed control diets (4.24%). It is likely that this magnitude of difference in marbling would elicit differences in meat palatability (O’Quinn et al., 2012; Corbin et al., 2015). Geng et al. (2016b) reported no differences in sensory juiciness or flavor between bulls fed active dry *S. cerevisiae* when compared with control samples. However, Geng et al. (2022) did report improved tenderness of beef from bulls supplemented with active dry *S. cerevisiae* compared with control samples. Overall, the present study concluded that limited differences in palatability of *longissimus thoracis* samples should be expected when steers were supplemented with benzoic acid, active dry *S. cerevisiae*, or a combination of the two. However, greater research efforts may be warranted on this topic due to conflicting findings from previous studies.

**Table 2.** Trained sensory panel analysis and instrumental tenderness of beef *longissimus thoracis* from steers fed a high-grain finishing diet with no supplementation, benzoic acid, active dry yeast, or a combination of benzoic acid and active dry yeast

	Treatment^1^					
Item	CON	ACD	YST	AY	SEM	*P*-value
Sensory analysis^2^						
Tenderness	8.71	8.73	8.05	8.09	0.58	0.10
Chewiness	6.35^b^	6.05^b^	6.73^ab^	7.51^a^	0.61	0.01
Juiciness	6.66	7.19	7.11	6.80	0.60	0.44
Beef flavor intensity	7.98	7.70	7.92	8.35	0.60	0.23
Off-flavor intensity	2.00	1.70	1.68	1.84	0.67	0.52
Instrumental tenderness^3^						
7-d *post-mortem* aging, kg	2.83	3.14	3.25	3.07	0.16	0.33
14-d *post-mortem* aging, kg	2.47	2.79	2.83	2.54	0.19	0.21

^a-b^ Least square means within a row with different superscripts differ (*P* < 0.05).

^1^ Treatments: CON (*n* = 15): control (not supplemented); ACD (*n* = 14): 0.5% of benzoic acid dietary inclusion on a DM basis (DSM Nutritional Products); YST (*n* = 15): 3g/hd/d of *Saccharomyces cerevisiae* (Vistacell, AB Vista, Marlborough, UK); AY (*n* = 13): 0.5% of benzoic acid dietary inclusion on a DM basis (DSM Nutritional Products) and 3 g/hd/d of *Saccharomyces cerevisiae* (Vistacell, AB Vista, Marlborough, UK).

^2^ Sensory traits were measured on a 15-cm line scale; tenderness: 0 = extremely tough to 15 = extremely tender; chewiness: 0 = not chewy to 15 = extremely chewy; juiciness: 0 = very little juiciness to 15 = very high juiciness; beef flavor intensity: 0 = very weak beef flavor detected to 15 = very intense beef flavor; off-flavor intensity: 0 = no off-flavors detected to 15 = very intense off-flavor.

^3^ Instrumental tenderness was measured using Warner–Bratzler shear force.

### Simulated Retail Display

**Figure 1.**
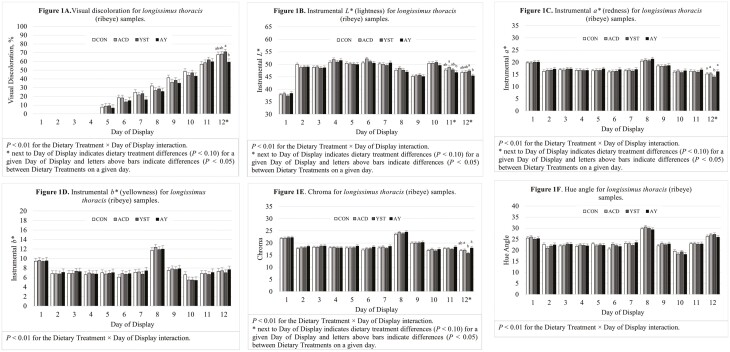
Visual discoloration (A) and instrumental color (L* in B, a* in C, b* in D, chroma in E, and hue angle in F) for beef *longissimus thoracis* during a simulated retail display period from steers fed a high-grain finishing diet with no supplementation (CON), benzoic acid (ACD), active dry yeast (YST), or a combination of benzoic acid and active dry yeast (AY).

**Figure 2.**
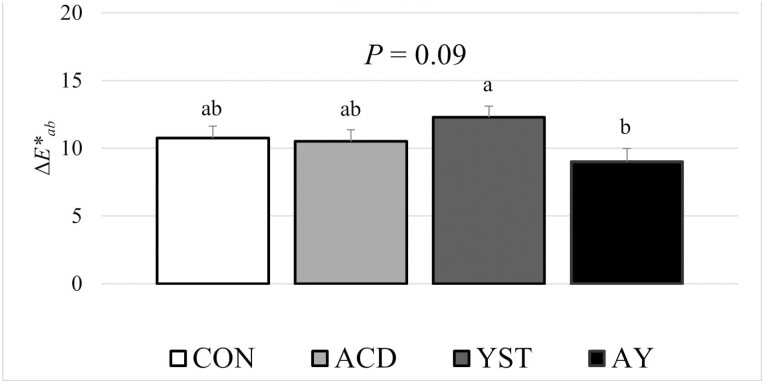
Total color change (ΔE*_ab_) for beef *longissimus thoracis* during a simulated retail display period from steers fed a high-grain finishing diet with no supplementation (CON), benzoic acid (ACD), active dry yeast (YST), or a combination of benzoic acid and active dry yeast (AY).

There was significant dietary treatment-by-day interactions (*P* < 0.01) for visual discoloration, L*, a*, b*, chroma, and hue angle during the simulated retail display period (Figure 1). Treatment differences (*P* < 0.05) were detected on day 12 of the simulated retail display period for visual discoloration, on days 11 and 12 of the simulated retail display period for L*, and on day 12 of the simulated retail display period for a* and chroma. There were no dietary treatment differences (*P* > 0.10) at any of the display days for b* and hue angle. On day 12 of the simulated retail display period, samples from steers supplemented with YST had greater levels of visual discoloration when compared with samples from steers supplemented with AY, while samples from steers fed CON and samples from steers supplemented with ACD were at intermediate values that were not different from the other treatments. These findings were confirmed with instrumental color values, where samples from steers fed AY had the lowest L* values on both days 11 and 12 of the simulated retail display period and the highest a* and chroma values on day 12 of the simulated retail display period. Total color change (Figure 2) may perhaps be an easier variable to interpret with the parameter tending to differ (*P* = 0.09) during the 12-d simulated retail display period with samples from steers supplemented with AY experiencing less color change compared with samples from steers supplemented with YST, while samples from steers fed CON and samples from steers supplemented with ACD were at intermediate values that were not different from the other dietary treatments. There has been limited work conducted previously on color stability of beef from cattle-fed organic acids or active dry yeast products. In the limited literature available, Wang et al. (2020b) reported no differences in color stability between *longissimus thoracis* samples from steers supplemented with benzoic acid when compared with steers fed control diets, while Geng et al. (2016a) reported no difference in initial color values for sirloin steak samples from bulls supplemented with active dry *S. cerevisiae* when compared with bulls fed control diets.

**Figure 3.**
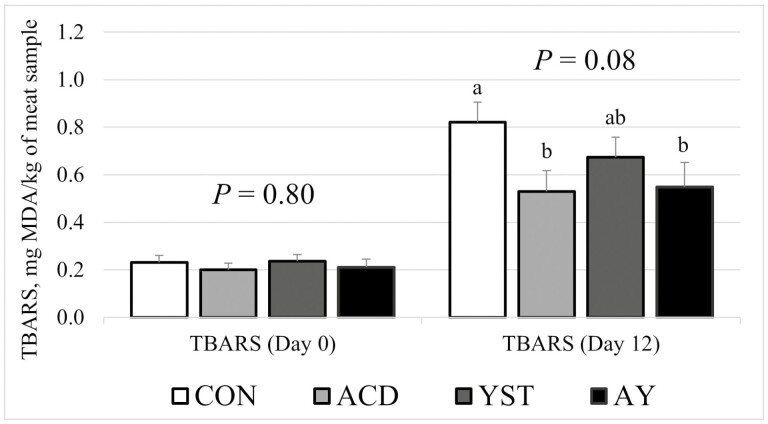
Thiobarbituric acid reactive substances (TBARS) for beef *longissimus thoracis* during a simulated retail display period from steers fed a high-grain finishing diet with no supplementation (CON), benzoic acid (ACD), active dry yeast (YST), or a combination of benzoic acid and active dry yeast (AY).

While there were no dietary treatment differences in lipid oxidation (as measured with TBARS) on day 0 (*P* = 0.80), lipid oxidation tended to differ (*P* = 0.08) following the 12-d simulated retail display period with samples from steers supplemented with ACD (0.53 mg MDA/kg of meat sample) or AY (0.55 mg MDA/kg of meat sample) experiencing lower levels of oxidation on day 12 compared with samples from steers fed CON (0.82 mg MDA/kg of meat sample), while samples from steers supplemented with YST (0.67 mg MDA/kg of meat sample) were at intermediate values that were not different from the other treatments (Figure 3). This is a meaningful finding that would suggest greater shelf-life stability for samples from steers supplemented with benzoic acid. However, the only study that has previously examined the effects of feeding benzoic acid on beef shelf life stability (Wang et al., 2020b) only partially supported this finding as TBARS values when expressed as mg MDA/kg of *longissimus thoracis* sample were not different, yet TBARS values when expressed as mg MDA/g lipid of *longissimus thoracis* sample were reduced in the samples from steers supplemented with benzoic acid when compared with the samples from steers not supplemented with benzoic acid. Thus, greater emphasis on oxidative stability of meat samples from cattle supplemented with benzoic acid is likely warranted. In addition, the mechanistic action for oxidative stability of beef products remains an area of research that should continue to be prioritized, especially in terms of pre-harvest effects such as the incorporation of novel dietary feed additives (Decker, 1998; Suman et al., 2014; Jiang and Xiong, 2016).

## Conclusions

Overall, these results suggest there were minimal negative effects on meat quality when finishing steers were supplemented with either benzoic acid and/or *S. cerevisiae*, and there may even be advantages for fatty acid composition and oxidative stability when steers were supplemented with benzoic acid. Future research should continue to investigate this line of research and place particular interest on the chemistry of meat color and the formation of anti-oxidative compounds in meat from cattle supplemented with benzoic acid.

## Supplementary Material

txac161_suppl_Supplementary_Tables
