# Supplementary material for: The effects of feeding benzoic acid and/or active dry yeast (Saccharomyces cerevisiae) on fatty acid composition, sensory attributes, and retail shelf-life of beef longissimus thoracis
Source: Transl Anim Sci. 2022 Dec 7;7(1):txac161. doi: 10.1093/tas/txac161 (PMC9825282; doi:10.1093/tas/txac161)
Supplement: txac161_suppl_Supplementary_Tables [file txac161_suppl_supplementary_tables.docx]

| **Supplementary Table 1.** Fatty acid profile (expressed in units of mg / 100 g sample) for beef *longissimus thoracis* from steers fed a high-grain finishing diet with no supplementation, benzoic acid, active dry yeast, or a combination of benzoic acid and active dry yeast. | | | | | | |
| --- | --- | --- | --- | --- | --- | --- |
|  | Treatment^1^ | | | |  |  |
| Item | CON | ACD | YST | AY | SEM | *P*-value |
| Fatty acid composition, mg / 100 g sample | | | | | | |
| C6:0 | 2.34 | 2.29 | 2.54 | 3.21 | 0.37 | 0.26 |
| C7:0 | 6.89 | 6.56 | 7.96 | 7.45 | 1.10 | 0.62 |
| C8:0 | 3.51 | 3.82 | 3.47 | 3.58 | 0.40 | 0.90 |
| C9:0 | 2.47 | 1.51 | 2.10 | 2.06 | 0.37 | 0.17 |
| C10:0 | 15.62 | 18.16 | 17.62 | 17.17 | 2.10 | 0.80 |
| C11:0 | 2.56 | 2.20 | 2.37 | 2.17 | 0.81 | 0.98 |
| C12:0 | 18.02 | 19.35 | 17.72 | 21.20 | 1.86 | 0.52 |
| C14:0 | 700.91 | 778.55 | 701.52 | 745.39 | 67.48 | 0.79 |
| C15:0 | 104.17 | 117.67 | 107.90 | 108.37 | 8.68 | 0.70 |
| C16:0 | 6,107.62 | 6,732.42 | 6,189.63 | 5,895.37 | 478.70 | 0.60 |
| C17:0 | 288.73 | 330.44 | 312.67 | 279.19 | 21.88 | 0.32 |
| C18:0 | 2,952.35 | 3,147.78 | 2,951.03 | 2,707.51 | 206.79 | 0.51 |
| C19:0 | 17.85 | 18.22 | 18.35 | 15.79 | 1.17 | 0.38 |
| C20:0 | 15.97 | 17.96 | 16.56 | 17.48 | 1.66 | 0.81 |
| C22:0 | 6.13 | 6.00 | 6.04 | 6.42 | 0.61 | 0.96 |
| C24:0 | 9.45 | 9.47 | 9.03 | 9.95 | 0.65 | 0.71 |
| C12:1 | 3.58 | 3.20 | 3.67 | 4.23 | 0.72 | 0.61 |
| C14:1 | 141.91 | 163.25 | 145.74 | 158.74 | 16.48 | 0.74 |
| C15:1 | 8.40 | 8.43 | 10.26 | 8.73 | 1.38 | 0.62 |
| C16:1-Trans | 35.78 | 36.07 | 35.00 | 37.43 | 2.69 | 0.91 |
| C16:1 | 674.45 | 735.83 | 734.14 | 710.98 | 61.82 | 0.84 |
| C17:1 | 200.53 | 215.53 | 221.56 | 204.15 | 19.57 | 0.84 |
| C18:1-Trans (Elaidate) | 144.01 | 153.28 | 130.08 | 107.90 | 13.23 | 0.09 |
| C18:1-Trans | 801.66 | 756.84 | 674.27 | 658.50 | 85.60 | 0.56 |
| C18:1 (Oleate) | 7,641.89 | 8,352.65 | 8,218.23 | 7,327.24 | 565.99 | 0.50 |
| C18:1 (Vaccenate) | 353.44 | 366.40 | 373.55 | 350.08 | 22.36 | 0.85 |
| C19:1 | 21.38 | 21.28 | 21.19 | 20.78 | 1.97 | 0.99 |
| C20:1-Cis-5 | 51.86 | 53.88 | 49.27 | 55.10 | 3.81 | 0.69 |
| C20:1-Cis-8 | 20.30 | 21.91 | 20.38 | 20.54 | 1.44 | 0.82 |
| C20:1-Cis-11 | 39.31 | 42.00 | 42.86 | 39.43 | 3.29 | 0.80 |
| C22:1 | 1.47 | 1.47 | 0.93 | 2.10 | 0.63 | 0.08 |
| C24:1 | 7.19^ab^ | 7.18^ab^ | 9.43^a^ | 5.99^b^ | 0.96 | 0.05 |
| C18:2 n6-Trans | 37.56 | 39.14 | 35.91 | 33.37 | 2.77 | 0.49 |
| C18:2 n6 | 582.17 | 487.51 | 568.31 | 530.16 | 42.19 | 0.35 |
| C18:3 n3 | 65.38 | 66.20 | 60.72 | 65.62 | 4.52 | 0.75 |
| C18:4 n3 | 19.47 | 23.88 | 20.56 | 21.24 | 2.62 | 0.64 |
| C20:2 n6 | 20.02 | 18.66 | 19.77 | 18.54 | 1.43 | 0.82 |
| C20:3 n6 | 41.97 | 39.70 | 41.32 | 38.78 | 1.44 | 0.35 |
| C20:3 n3 | 5.11 | 5.41 | 5.15 | 5.42 | 0.83 | 0.98 |
| C20:4 n6 | 112.60^a^ | 93.90^b^ | 108.58^a^ | 105.66^ab^ | 5.13 | 0.05 |
| C20:4 n3 | 5.18 | 5.58 | 5.62 | 5.92 | 0.81 | 0.93 |
| C20:5 n3 | 14.50 | 15.22 | 15.13 | 16.18 | 0.98 | 0.67 |
| C22:4 n6 | 18.05 | 17.45 | 19.27 | 16.73 | 1.05 | 0.34 |
| C22:5 n6 | 5.91 | 4.82 | 6.03 | 5.19 | 0.81 | 0.68 |
| C22:5 n3 | 44.84 | 46.46 | 43.91 | 48.10 | 1.53 | 0.21 |
| C22:6 n3 | 6.88 | 6.01 | 5.73 | 5.49 | 0.63 | 0.23 |
| ^a-b^ Least square means within a row with different superscripts differ (*P* < 0.05).  ^1^ Treatments: CON (n=15): control (not supplemented); ACD (n=14): 0.5% of benzoic acid dietary inclusion on a DM basis (DSM Nutritional Products); YST (n=15): 3g/hd/d of *Saccharomyces cerevisiae* (Vistacell, AB Vista, Marlborough, UK); AY (n=13): 0.5% of benzoic acid dietary inclusion on a DM basis (DSM Nutritional Products) and 3g/hd/d of *Saccharomyces cerevisiae* (Vistacell, AB Vista, Marlborough, UK). | | | | | | |

| **Supplementary Table 2.** Fatty acid profile (expressed in units of % of total fatty acid) for beef *longissimus thoracis* from steers fed a high-grain finishing diet with no supplementation, benzoic acid, active dry yeast, or a combination of benzoic acid and active dry yeast. | | | | | | |
| --- | --- | --- | --- | --- | --- | --- |
|  | Treatment^1^ | | | |  |  |
| Item | CON | ACD | YST | AY | SEM | *P*-value |
| Fatty acid composition, % of total fatty acids | | | | | | |
| C6:0 | 0.01 | 0.01 | 0.01 | 0.01 | 0.002 | 0.06 |
| C7:0 | 0.03 | 0.03 | 0.04 | 0.04 | 0.004 | 0.37 |
| C8:0 | 0.02 | 0.02 | 0.02 | 0.02 | 0.002 | 0.80 |
| C9:0 | 0.01 | 0.01 | 0.01 | 0.01 | 0.002 | 0.79 |
| C10:0 | 0.07 | 0.08 | 0.08 | 0.08 | 0.007 | 0.83 |
| C11:0 | 0.01 | 0.01 | 0.01 | 0.01 | 0.003 | 0.91 |
| C12:0 | 0.08 | 0.09 | 0.08 | 0.10 | 0.005 | 0.07 |
| C14:0 | 3.23 | 3.35 | 3.17 | 3.54 | 0.155 | 0.32 |
| C15:0 | 0.48 | 0.51 | 0.49 | 0.53 | 0.020 | 0.43 |
| C16:0 | 28.43 | 29.04 | 28.11 | 28.66 | 0.523 | 0.58 |
| C17:0 | 1.35 | 1.44 | 1.42 | 1.38 | 0.044 | 0.23 |
| C18:0 | 13.85 | 13.57 | 13.46 | 13.33 | 0.303 | 0.63 |
| C19:0 | 0.08 | 0.08 | 0.08 | 0.08 | 0.004 | 0.58 |
| C20:0 | 0.08 | 0.08 | 0.08 | 0.09 | 0.007 | 0.43 |
| C22:0 | 0.03 | 0.03 | 0.03 | 0.03 | 0.004 | 0.90 |
| C24:0 | 0.05 | 0.04 | 0.04 | 0.05 | 0.004 | 0.53 |
| C12:1 | 0.02 | 0.01 | 0.02 | 0.02 | 0.003 | 0.12 |
| C14:1 | 0.65 | 0.71 | 0.65 | 0.76 | 0.051 | 0.36 |
| C15:1 | 0.04 | 0.04 | 0.05 | 0.04 | 0.004 | 0.33 |
| C16:1-Trans | 0.17^b^ | 0.16^b^ | 0.16^b^ | 0.18^a^ | 0.005 | 0.01 |
| C16:1 | 3.12 | 3.22 | 3.31 | 3.40 | 0.131 | 0.40 |
| C17:1 | 0.93 | 0.94 | 0.99 | 1.00 | 0.058 | 0.75 |
| C18:1-Trans (Elaidate) | 0.68 | 0.67 | 0.59 | 0.52 | 0.045 | 0.07 |
| C18:1-Trans | 3.84 | 3.30 | 3.15 | 3.22 | 0.374 | 0.50 |
| C18:1 (Oleate) | 35.68 | 36.40 | 37.09 | 35.94 | 0.720 | 0.48 |
| C18:1 (Vaccenate) | 1.67 | 1.61 | 1.72 | 1.71 | 0.048 | 0.35 |
| C19:1 | 0.10 | 0.09 | 0.09 | 0.10 | 0.005 | 0.44 |
| C20:1-Cis-5 | 0.24^ab^ | 0.24^b^ | 0.23^b^ | 0.27^a^ | 0.012 | 0.03 |
| C20:1-Cis-8 | 0.10 | 0.10 | 0.09 | 0.10 | 0.003 | 0.08 |
| C20:1-Cis-11 | 0.19 | 0.19 | 0.19 | 0.20 | 0.015 | 0.91 |
| C24:1 | 0.03 | 0.04 | 0.03 | 0.03 | 0.004 | 0.09 |
| C18:2 n6-Trans | 0.18 | 0.17 | 0.17 | 0.16 | 0.007 | 0.55 |
| C18:2 n6 | 2.83 | 2.21 | 2.71 | 2.64 | 0.231 | 0.23 |
| C18:3 n3 | 0.31 | 0.30 | 0.28 | 0.32 | 0.016 | 0.22 |
| C18:4 n3 | 0.09 | 0.10 | 0.09 | 0.10 | 0.008 | 0.55 |
| C20:2 n6 | 0.09 | 0.08 | 0.09 | 0.09 | 0.007 | 0.51 |
| C20:3 n6 | 0.20 | 0.18 | 0.20 | 0.20 | 0.013 | 0.55 |
| C20:3 n3 | 0.02 | 0.02 | 0.02 | 0.03 | 0.005 | 0.68 |
| C20:4 n6 | 0.55 | 0.43 | 0.53 | 0.54 | 0.047 | 0.18 |
| C20:4 n3 | 0.02 | 0.02 | 0.03 | 0.03 | 0.004 | 0.75 |
| C20:5 n3 | 0.07 | 0.07 | 0.07 | 0.08 | 0.008 | 0.63 |
| C22:4 n6 | 0.09 | 0.08 | 0.09 | 0.09 | 0.008 | 0.61 |
| C22:5 n6 | 0.03 | 0.03 | 0.03 | 0.03 | 0.004 | 0.87 |
| C22:5 n3 | 0.22 | 0.22 | 0.21 | 0.25 | 0.017 | 0.44 |
| C22:6 n3 | 0.03 | 0.03 | 0.03 | 0.03 | 0.004 | 0.51 |
| ^a-b^ Least square means within a row with different superscripts differ (*P* < 0.05).  ^1^ Treatments: CON (n=15): control (not supplemented); ACD (n=14): 0.5% of benzoic acid dietary inclusion on a DM basis (DSM Nutritional Products); YST (n=15): 3g/hd/d of *Saccharomyces cerevisiae* (Vistacell, AB Vista, Marlborough, UK); AY (n=13): 0.5% of benzoic acid dietary inclusion on a DM basis (DSM Nutritional Products) and 3g/hd/d of *Saccharomyces cerevisiae* (Vistacell, AB Vista, Marlborough, UK). | | | | | | |
